# Use of the Stingray Re-Entry System in Two Complex Cases of Occluded Superficial Femoral Arteries

**DOI:** 10.1155/2011/976312

**Published:** 2011-09-14

**Authors:** Erin M. Galbraith, Marc Del Rosario, Khusrow Niazi

**Affiliations:** Division of Cardiology, Department of Internal Medicine, Emory University, Atlanta, GA 30312, USA

## Abstract

Totally occluded infrainguinal arterial disease presents formidable challenges to endovascular revascularization. A variety of devices have been made available to make the crossing of these lesions more amenable to endovascular techniques. We discuss the novel use of a device that has been developed for crossing occluded coronary arteries, the Stingray Re-Entry System.

## 1. Introduction

**Figure 1:**
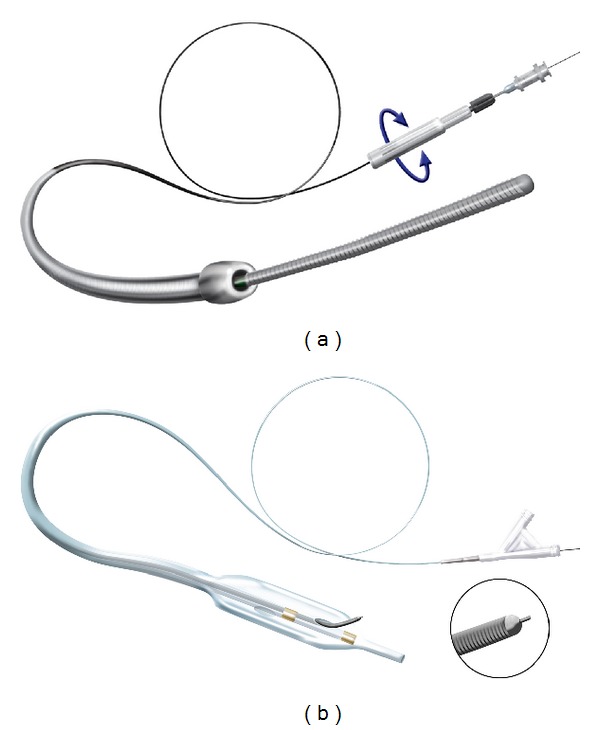
The CrossBoss and Stingray catheters. (a) The CrossBoss catheter is an over-the-wire device with a 1 mm rounded tip, a coiled shaft and a moveable proximal torque device that is released under high torque to prevent product damage. (b) The Stingray orienting balloon catheter is an over-the-wire, 10 mm long, 2.5 mm wide, flat balloon catheter designed to deploy within the subintimal plane with exit ports on either side of the balloon. Marker bands identify the location of the exit ports. The Stingray guidewire has a 28-degree bend and a distal probe that extends from the tip to aid reentry.

Endovascular treatment for peripheral arterial disease is more often being considered as the first line of treatment in patients needing revascularization. In infra-inguinal disease totally occluded vessels present formidable challenges including an increased risk to the patient. Over the past few years a variety of devices have been made available to make the crossing of these lesions more amenable to endovascular techniques. In the following two cases we describe the use of a novel device that has been developed for crossing occluded coronary arteries. The Stingray Re-Entry System (Bridge-Point Medical, Plymouth, Minn) has been recently reported in crossing tibial vessels [1] and coronary chronic occlusions [2, 3], but its use in the superficial femoral artery (SFA) has not been reported (Figures 1(a) and 1(b)).

## 2. Case Reports

### 2.1. Case 1

A 77-year-old woman with hypertension and former tobacco use presented in March 2010 with a two-year history of progressive bilateral thigh Rutherford-Becker class 3 claudication despite medication optimization. Ankle-brachial indices were 0.65 for the right leg with monophasic wave forms and 0.86 for the left with biphasic wave forms. Angiography revealed no significant arterial calcification with a 60% stenosis of the mid-right common iliac artery with no pressure gradient and a 100% ostial right SFA stenosis with reconstitution of the distal right SFA via collateral blood flow from the deep femoral artery. The posterior tibial artery provided 1-vessel runoff to the right foot. The left lower extremity angiogram was significant for a 30–40% stenosis in the mid-SFA and an occluded mid-anterior tibial artery that reconstituted distally via collateral flow with 2-vessel runoff to the left foot.

**Figure 2:**
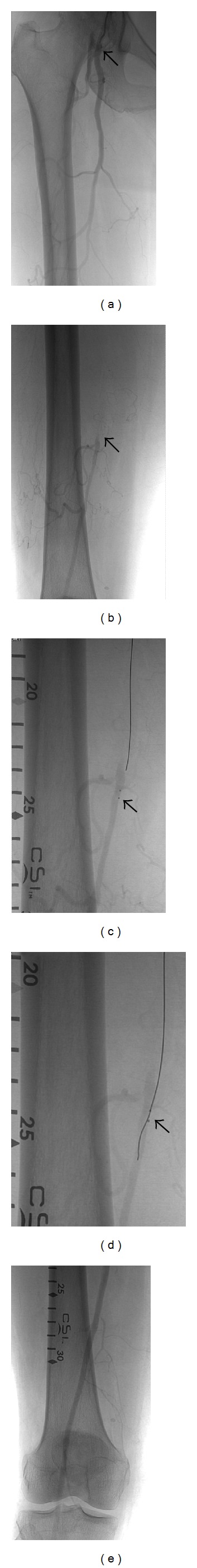
Successful use of the CrossBoss and Stingray catheter. (a) Angiography showing occlusion of the right superficial femoral artery (SFA) at its ostium (arrow). (b) Angiography showing the distal SFA reconstituted by collateral flow from the right profunda. (c) Wire inside the StingRay balloon catheter (arrow). The catheter is within the subintimal space distal to the SFA occlusion. The two radio-opaque dots are the two exit ports on the catheter. (d) Wire advanced through the StingRay balloon catheter (arrow) exiting into the true lumen of the distal SFA. (e) Postinterventional final angiogram revealing antegrade flow through the previously occluded SFA.

The patient was brought back a few weeks later for percutaneous revascularization. Retrograde access of the left common femoral artery was obtained and a 7 F 45 cm sheath was advanced over the aortic bifurcation into the right common femoral artery. The CrossBoss (Bridge-Point Medical, Plymouth, MN) catheter was advanced through the sheath to the SFA ostium, and the rotational tip was advanced through the SFA occlusion to the popliteal artery. As the tip of the catheter was subintimal, the catheter was exchanged out for the Stingray Re-Entry System catheter over a Miracle wire (Ashai Intec, Seto, Japan). The Stingray Re-Entry System balloon was dilated to 4 atmospheres to stabilize the catheter in the subintima of the popliteal artery. The dedicated Stingray Re-Entry System guide wire advanced through the catheter and exited the distal port of the balloon catheter as designed, penetrating the intima into the true lumen of the popliteal artery. Subsequently, the patient underwent angioplasty and atherectomy after placement of a distal protection device. Right iliac angiogram with runoff postintervention was performed with digital subtraction imaging revealing good angiographic results (Figure 2).

### 2.2. Case 2

An 81-year-old male with a history of hypertension, diabetes mellitus, and chronic kidney disease stage 3, was referred by his primary care physician for worsening Rutherford-Becker class 3 claudication of both calves despite medication optimization. Ankle-brachial indices were 0.60 in both lower extremities with monophasic waveforms. Magnetic resonance angiography of his lower extremities showed severe ostial right SFA stenosis and diffuse moderate disease distally. The left SFA had a subtotal occlusion at its origin with reconstitution of mid-to distal segments. The left foot had one-vessel runoff by the peroneal artery. The patient was taken to the catheterization laboratory for possible percutaneous atherectomy of the left SFA. 

**Figure 3:**
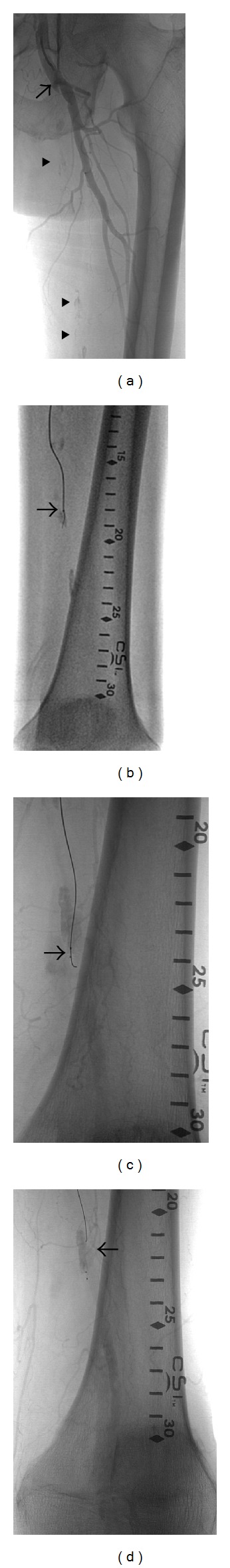
Unsuccessful use of the CrossBoss and Stingray catheter. (a) Angiography showing occlusion of the left superficial femoral artery (SFA) at its ostium (arrow). The course of the occluded vessel is outlined by areas of calcification (arrowheads). (b) Wire inside the CrossBoss catheter (arrow) crossing an occluded, calcified segment. (c) Wire advanced through the StingRay balloon catheter (arrow) trying to gain entry into the true lumen of the distal SFA. (d) Wire back into the StingRay balloon catheter with final angiography showing retrograde filling of the distal SFA and some contrast extravasation (arrowhead).

Arterial access was obtained at the contralateral femoral artery and a 7 F 45 cm sheath was used. Angiography showed 100% ostial stenosis of the left SFA with reconstitution of the distal third of the vessel via collaterals from the profunda femoris artery. A 6 F multipurpose A2 (MPA2) guide catheter was advanced in the sheath and positioned at the ostium of the left SFA. The CrossBoss (Bridge-Point Medical, Plymouth, MN) catheter was advanced through the MPA2, and the rotational tip was advanced through the SFA occlusion. A High-Torque Ironman Guidewire (Abbott Vascular, Redwood City, Calif), and later an Asahi Confianza Pro Guidewire (Abbott Vascular, Redwood City, CA) were used to steer and support the catheter. Multiple calcified areas along the course of the artery caused difficulty in advancing the catheter. With careful manipulation, the CrossBoss catheter tip successfully reached the distal third of the vessel after running a subintimal course. The catheter was exchanged out for the Stingray Re-Entry System catheter. The Stingray Re-Entry balloon was inflated to stabilize its position. Despite repeated efforts with the dedicated Stingray guidewire, and later a Runthrough NS 180 cm wire (Terumo Cardiovascular Systems, Ann Arbor, Mchi), access into the true lumen of the distal SFA could not be obtained. The procedure was then aborted (Figure 3).

## 3. Discussion

These two complex cases of totally occluded SFA show the challenges that confront endovascular therapeutics. The first case had no discernible calcification on angiography whereas the second case demonstrated moderate to severe calcification. The Stingray Re-Entry System was successful in accessing the true lumen after crossing the occluded vessel in the first case and one of the discernible differences being lack of detectable calcification.

One of the advantages of the Stingray Re-Entry System is its low profile which avoids creating a large subintimal space unlike some of the currently available devices. The low profile may also reduce the risk of distal embolization. Another advantage may be its lack of complex hardware or need for intravascular ultrasound which may impact the financial cost of the procedure. Also there are no sharp needles for reentry. However, since the system has been developed for the coronary arteries, it may need to be modified for use in the larger vessels like the femoral and popliteal arteries where having a larger balloon with the ability to use a 0.018 inch wire may allow calcified arteries to be addressed successfully.

Finally, before this device is considered a go-to device in totally occluded vessels, a randomized trial comparing it to the conventional method of a wire and catheter in totally occluded vessels would be most helpful.
